# Biological Properties of a Citral-Enriched Fraction of *Citrus limon* Essential Oil

**DOI:** 10.3390/foods9091290

**Published:** 2020-09-14

**Authors:** Marzia Pucci, Stefania Raimondo, Chiara Zichittella, Vincenza Tinnirello, Valeria Corleone, Gioacchino Aiello, Marta Moschetti, Alice Conigliaro, Simona Fontana, Riccardo Alessandro

**Affiliations:** 1 Department of Biomedicine, Neuroscience and Advanced Diagnostics (Bi.N.D), Section of Biology and Genetics, University of Palermo, 90133 Palermo, Italy; marzia.pucci@unipa.it (M.P.); stefania.raimondo@unipa.it (S.R.); zichy95.cz@gmail.com (C.Z.); marta.moschetti@unipa.it (M.M.); alice.conigliaro@unipa.it (A.C.); riccardo.alessandro@unipa.it (R.A.); 2 Agrumaria Corleone s.p.a., Via S. Corleone, 12-Zona Ind. Brancaccio, 90124 Palermo, Italy; vincenzatinni@gmail.com (V.T.); vacorleone@agrumariacorleone.com (V.C.); gaiello@agrumariacorleone.com (G.A.)

**Keywords:** *Citrus limon* (L.) Osbeck, essential oil, citral, nutraceuticals, inflammation, oxidative stress

## Abstract

Lemon essential oil (LEO) is a well-known flavoring agent with versatile biological activities. In the present study, we have isolated and characterized four citral-enriched fractions of winter LEO. We reported that in murine and human macrophages the pre-treatment with a mix of these fractions (Cfr-LEO) reduces the expression of the pro-inflammatory cytokines TNF-α, IL-1β, and IL-6 induced by LPS. In addition, Cfr-LEO counteracts LPS-induced oxidative stress, as shown by the increase in the GSH/GSSG ratio in comparison to cells treated with LPS alone. Overall, the results reported here encourage the application of EO fractions, enriched in citral, in the nutraceutical industry, not only for its organoleptic properties but also for its protective action against inflammation and oxidative stress.

## 1. Introduction

Essential oils (EOs), also called “volatile odoriferous oil”, are concentrated aromatic liquids of a combination of volatile compounds that can be extracted from different parts of the plant, for example, leaves, peels, barks, flowers, buds, seeds, twigs, and roots. Different isolation methods have been described, among which steam distillation [1,2,3]. 

The genus *Citrus*, one of the most important taxonomic subunits of the family *Rutaceae*, includes approximately 17 species of plants that produce some of the most cultivated fruits in the world. Among them, considering their significative content, the species most used for EO production are lemons (*Citrus limon* (L.) Osbeck), oranges (*Citrus sinensis* (L.) Osbeck), grapefruits (*Citrus paradisi* Macfad), both mandarins and tangerines (*Citrus reticulata* Blanco), and various limes (mainly *Citrus Aurantifolia* (Christm.) Swingle) [4]. The main components of the *C. limon* EO (LEO) are represented by monoterpenoids [5,6,7]. In particular, LEO is a complex mixture of limonene, γ-terpinene, citral, linalool, β-caryophyllene, α-pinene, and β-pinene [8]. 

Linalool, β-caryophyllene, and limonene possess anti-inflammatory effects [9,10,11]; instead, α-pinene and β-pinene exert antioxidant effects by reducing nitric oxide production [12]. 

Moreover, citral, a mixture of the two aldehydes geranial and neral, which represents one of the main bioactive components of lemon oil (65–85%), is known to possess various medicinal properties as inhibiting oxidant activity, nuclear factor kappa B (NF-kB) activation, and cyclooxygenase-2 (COX-2) expression. Katsukawa M. et al., showed that citral, in human macrophage-like U937 cells, induces expression of PPARα and -γ responsive genes and suppressed both LPS-induced COX-2 mRNA and protein expression in a PPARγ-dependent manner [13]. Bouzenna H. et al. showed antioxidant effects of citral in rat small intestine epithelial cells, suggesting that citral can protect against aspirin-induced oxidative stress [14]. Interestingly, citral also has a significant effect in cancer prevention and treatment [15]; for example, hepatocarcinogenesis in rats was inhibited by lemongrass oil with a high content of citral [16]. Belusamy et al. also demonstrated that citral inhibited cell viability, proliferation, and clonogenic potential of prostate cancer cells by targeting key players of fatty acid biosynthesis [17].

Furthermore, citral mainly contributes to lemon flavor and for this reason, it is usually applied in the nutraceutical industry, which today is extremely interested in finding innovative solutions to obtain plant derivates with beneficial properties, to add to their existing products like food, beverages, and cosmetics.

Increasing population aging is unfortunately associated with the occurrence of age-related and inflammatory-based disorders. The relationship between oxidative stress and chronic inflammation is well known from the literature; in particular, reactive oxygen species (ROS) disrupts cell signaling, alters the metabolism of arachidonic acid, promotes or enhances airway and systemic inflammation [18]. Among inflammatory and immune effector cell types, macrophages play a crucial role in the immune response, producing both pro-inflammatory cytokines and other inflammatory mediators [19], for example, tumor necrosis factor α (TNF-α), interleukin-1β (IL-1β), IL-6. To date, pro-inflammatory and pro-oxidant regulators are considered important targets for the development of therapeutic strategies. 

Different studies focused their attention on developing inhibitors from natural resources to prevent or alleviate chronic inflammatory conditions that can be used with minimal side effects. 

According to the literature, LEO has versatile therapeutic activities on the digestive apparatus and the cardiovascular, nervous, and immune systems [20,21,22,23,24,25,26,27,28,29,30,31]. To date, most studies are centered on the functional analysis of whole LEO, highlighting its antioxidant and anti-inflammatory properties specifically related to the presence of linalool [11] and limonene [20]. Less consideration has been given to the biological properties of the fractions obtained from whole oil; in fact, the few available information is mainly focused on the isolation techniques and the molecular profiling description [32,33,34,35,36], rather than on the functional role of the fractions [37,38,39]. 

This study aimed to evaluate the anti-inflammatory and antioxidant activity of citral-enriched fractions of *C. limon* EO (Cfr-LEO) that, for their improved aroma profile, could have great commercial importance as natural food additives. With this purpose, *C. limon* EO was obtained by cold-pressed extraction from winter fruit and the Cfr-LEO isolated by chromatographic technique. The effects of Cfr-LEO were investigated on LPS-stimulated murine and human macrophages.

## 2. Materials and Methods

### 2.1. Purification of Enriched Fractions from *Citrus limon* L. Essential Oil

*C. limon* essential oil (LEO) was recovered by cold-pressed extraction mechanical process from the peels of winter fruits at the company Agrumaria Corleone S.P.A. (Palermo, Italy). 

After cold dewaxing at −20 °C for 48 h and subsequent filtration through a paper filter with 10-micron pores, LEO was fractionated by a newly developed adsorption column chromatography. Some volumes of essential oil flowed through the chromatographic column filled with a particular type of stationary phase under the following operating conditions: pressure: atmospheric; temperature: 25 °C; flow: 3 mL/min. This newly developed method allowed the collections of fractions enriched with the main aromatic compounds present in *C. limon* essential oil, based on their affinity with the stationary phase. Other technical details cannot here be reported and described since to date are covered by trade secrets.

### 2.2. Gas Chromatography (GC-MS and GC-FID) Analyses

The composition of volatile constituents of the essential oil was analyzed with Agilent 6890 N Network gas chromatographic (GC) equipped with Agilent 5973 mass spectrometer (Agilent Technologies Inc., Santa Clara, CA, USA) with a DB-5MS fused silica column (30 m × 0.25 mm ID, film thickness 0.25 μm, Agilent Technologies Inc., Saint Clara, CA, USA). The oven program started with an initial temperature of 75 °C held for 9 min, then the oven temperature was heated at 4 °C/min to 120 °C and after at 5 °C/min to 310 °C for 15 min. The source temperature was 230 °C, quadrupole temperature was 150 °C, injector and detector were 250 and 280 °C, respectively. The carrier gas was helium adjusted to a linear velocity of 42 cm/s. Samples were prepared by diluting in hexane in a ratio of 1:10. Samples were injected (0.2 μL) with a split ratio of 1:50. The identification of essential oil volatile components was performed by comparison of their mass spectra with a NIST MS Search and Wiley 138 mass spectral library, as well as with literature data. 

The GC-FID analysis of the EO was performed with GC Agilent 7890 A (Agilent Technologies Inc., Santa Clara, CA, USA) equipped with a non-polar DB-5 (Agilent Technologies Inc., Santa Clara, CA, USA) capillary column (length: 20 m; 100 μm internal diameter; film thickness 0.1μm). The oven temperature program was set to 75 °C for 3 min, 8 °C/min to 140 °C for 0 min, 30 °C/min to 310 °C for 5 min. The carrier gas was H2, at 0.80 mL/min flow. Injector and detector temperatures were set at 280 and 350 °C, respectively. Each EO sample was prepared by diluting it in isooctane in a ratio of 1:10. The injection volume was set at 0.2 μL. The GC-FID analysis of the samples allowed to obtain the relative percentage quantity of the single components contained in the analyzed samples. The value of each analyte is expressed as the percentage area of the peak with respect to the total composition of the EO obtained from the GC-FID analysis.

### 2.3. Cell Culture and Treatment

The murine macrophage RAW264.7 cell line was purchased from ATCC (Manassas, VA, USA). Cells were cultured in Dulbecco’s modified Eagle’s medium (DMEM) supplemented with 10% Fetal Bovine Serum (FBS, Euroclone, UK), 2 mM  l-glutamine, 100 U/mL penicillin, and 100 μg/mL streptomycin (Euroclone, UK). The human monocyte THP-1 cell line was purchased from ATCC (Manassas, VA, USA). Cells were cultures in RPMI-1640 medium (Euroclone, UK) supplemented with 10% FBS, 2 mM L-glutamine, 100 U/mL penicillin, and 100 μg/mL streptomycin (Euroclone, UK). THP-1 monocytes were differentiated into M0 macrophages (THP-1 M0) as previously described [40]. In particular, cells were plated at 1 × 10^5^ cells/mL and incubated at 37 °C with 5% CO_2_ for 48 h in the presence of 50 ng/mL of Phorbol 12-myristate 13-acetate (PMA, Sigma-Aldrich, Saint Luis, MO, USA); subsequently, the conditioned medium containing PMA was removed and replaced with fresh medium for 3 days for cell recovering. Cells were then treated at different time points with various concentrations of LEO or Cfr-LEO, previously diluted in a solution consisting of 95% FBS and 5% DMSO. The biological assays reported in this manuscript have been carried out with 2 different batches of Cfr-LEO.

### 2.4. MTT (3-[4,5-Dimethylthiazol-2-yl]-2,5 Diphenyl Tetrazolium Bromide) Assay

Cell viability was determined by MTT assay as previously described [41]. RAW264.7 were seeded in triplicate at 3 × 10^3^ cells per well in 96-well plates; 24 h post-seeding, cells were treated for 24 and 48 h with different concentrations of LEO or Cfr-LEO (0.005%, 0.01%, 0.02%, 0.05%). THP-1 M0 were seeded in triplicate at 2 × 10^4^ cells per well in 48-well plates and treated for 24 and 48 h with LEO or Cfr-LEO (0.005%, 0.01%, 0.02%, 0.05%). The absorbance was measured by ELISA reader at 540 nm (Microplate Reader, BioTek, Winooski, VT, USA). Values are expressed as a percentage of cell growth versus control (untreated cells). 

### 2.5. Measurement of Cytotoxicity in Cells Exposed to Cfr-LEO Treatment

For the detection of cytotoxicity, the CellTox™ Green Cytotoxicity Assay (Catalog number G8741, Promega, Madison, WI, USA) was used. RAW264.7 were cultured in triplicate at 5 × 10^3^ cells per well into white-walled, opaque assay 96 well plates; 24h post-seeding, cells were treated for 24 and 48 h with different concentrations of LEO or Cfr-LEO (0.005%, 0.01%, 0.02%, 0.05%). THP-1 M0 cells were seeded in triplicate at 1 × 10^4^ cells per well into white-walled, opaque assay 96 well plates and treated for 24 and 48 h with LEO or Cfr-LEO (0.005%, 0.01%, 0.02%, 0.05%). Changes in membrane integrity that occur as a result of cell death were measured by the CellTox™ Green Cytotoxicity Assay following the manufactures instructions. The fluorescence, proportional to cytotoxicity, was measured by Glomax (Promega).

### 2.6. RNA Isolation and Real-Time PCR

Levels of IL1*β*, IL6, and TNF*α* were measured by Real-time PCR. RAW264.7 cells were seeded at 5 × 10^4^ cells per well in 12-well plates; 24 h after seeding, cells were treated for 2 h with different concentrations of LEO or Cfr-LEO (0.005%, 0.01%, 0.02%) and then exposed to LPS (500 ng/mL) for 6 h, without oil removal. THP-1 M0 cells were seeded at 1 × 10^5^ cells per well in 12-well plates and treated for 2 h with LEO or Cfr-LEO (0.005%, 0.01%) before their exposure to LPS (1 μg/mL) for 6 h, without oil removal. Levels of BAX, BAD, and BCL-2 were measured by Real-Time PCR in RAW264.7 cells treated with 0.01% and 0.02% of Cfr-LEO for 24 and 48 h. RNA was isolated using the commercially available Illustra RNA spin Mini Isolation Kit (GE Healthcare, Little Chalfont, Buckinghamshire, UK), according to manufacturer’s instructions. Total RNA from RAW264.7 or human THP-1 M0 cells was reverse transcribed to cDNA using the High Capacity cDNA Reverse Transcription kit (Applied Biosystems, Foster City, CA, USA). For quantitative SYBR Green Real-time PCR, the reaction was carried out in a total volume of 20 µL containing 2X SYBR Green I Master Mix (Applied Biosystems), 2 µL of cDNA, and 300 nM forward and reverse primers. The oligonucleotides used are reported in the Table 1.

Real-time PCR was performed in 48-well plates using the Step-One Real-Time PCR System (Applied Biosystems). Relative changes in gene expression between control and treated samples were determined using the ∆∆Ct method. Levels of the target transcript were normalized to a GAPDH endogenous control, constantly expressed in all samples (∆Ct). For ∆∆Ct values, additional subtractions were performed between treated samples and control ∆Ct values. Final values were expressed as fold change.

### 2.7. Enzyme Linked ImmunoSorbent Assay (ELISA) Assays

The amounts of IL6 and TNFα in culture supernatants were determined by using mouse IL6- and TNFα- specific ELISA kits (Thermo Fisher Scientific, Waltham, MA USA). 

RAW264.7 cells were seeded at 5 × 10^4^ cells per well in 12-well plates; 24 h after seeding, cells were treated for 2 h with 0.01% of Cfr-LEO and then exposed to LPS (500 ng/mL) for 6 h, without oil removal. At the end of the experimental time, the conditioned medium was collected and centrifuged to remove cellular debris. The ELISA assays were then performed according to the manufacturer’s instructions. 

### 2.8. Measurement of GSH and GSSG in Cells Exposed to Cfr-LEO Treatment

To detect and quantify total glutathione (GSH + GSSG), GSSG and GSH-to-GSSG ratios in THP-1 M0 cells treated with Cfr-LEO, the bioluminescent GSH/GSSG-Glo Assay kit (Catalog number V6611, Promega, Madison, WI, USA) was used. Cells were seeded at 1 × 104 cells per well into white-walled, opaque assay 96 well plates, treated for 2 h with Cfr-LEO (0.005% and 0.01%), and then exposed to LPS (1 μg/mL) for 6 h, without oil removal. After incubation, the bioluminescent GSH/GSSG-Glo Assay kit was used following the manufacturer’s instructions. Cells were lysed by shaking with an equal volume of either total or oxidized glutathione reagent for 5 min, and then, cell lysates were incubated for 30 min with 50 µL of luciferin generation reagent. After the addition of 100 µL of luciferin detection reagent, plates were equilibrated for 15 min at room temperature. The luminescence (net relative luminescence unit, RLU) was measured by Glomax (Promega). The results were expressed as the GSH/GSSG ratio, calculated as follows: GSH/GSSG ratio = (Net total glutathione RLU−Net GSSG RLU)/(Net GSSG RLU/2).

### 2.9. Statistical Analysis

Data are represented as means ± SD. Comparisons were made using a Student’s *t*-test. Values were considered statistically significant when *p* ≤ 0.05.

## 3. Results and Discussion

### 3.1. Characterization of the Fractions Isolated from Citrus limon EO

To exploit at best the use of LEO as a flavoring agent, an aim is to obtain fractions enriched in compounds responsible for the lemon aroma. With this purpose, within this study, starting from the whole LEO extracted from winter fruits, we isolated four citral-enriched fractions with an enhanced lemon flavor.

Overall, 43 compounds, representing more than 99% of the total volatiles, were analyzed by GC-FID in the whole LEO and in each fraction (Fr). These compounds are listed in Table 2 where the corresponding Retention time and the % are reported for whole LEO and for 4 out of the 16 isolated fractions, Fr13, Fr 14, Fr15, and Fr16. These fractions were selected since they were the only in which neral and geranial (n 26 and 28 in Table 2), the two isomers of citral considered the essence of the lemon aroma, were enriched. 

Table 3 summarizes the percentage trend of the main chemical classes of EOs (Monoterpenes, Aldehydes, Esters, Sesquiterpenes, Aliphatic Alcohols, Sesquiterpene Alcohols). Reported data show that within the class of aldehydes, the citral (a mixture of geranial and neral) is the major constituent and has an increment of about 30% in each of the four selected fractions in comparison to whole LEO; no differences were revealed for Monoterpenes, Sesquiterpenes, Aliphatic Alcohols, and Sesquiterpene Alcohols while there was a decrement of Esters. Internal olfactory panel tests have demonstrated that the fractions Fr13, Fr14, Fr15, and Fr16 enhanced their aroma properties in comparison to whole LEO, thus indicating that the 30% increase of citral determinates better olfactory characteristics.

We found that among the three technical replicates of the fractionation process, the percentage of compounds showing a coefficient of variation (CV%) ≤ 5 was 70% for Fr13, 72% for Fr14, 55.8% for Fr15, and 72% for Fr16 (Figure S1a) and in all fractions, CV% higher than 20% was not found for any compound. Overall, these results highlighted that the fractionation process used in this study ensures high reproducibility. Moreover, as reported in Figure S1b, among the four selected fractions, the percentage of the compounds including citral (specifically reported in Figure S1c) was comparable. Thus, according to the equivalent composition of these fractions, we decided to mix them to obtain a citral-enriched fraction of *C. limon* EO (Cfr-LEO), characterized by an increase in the aromatic level. In consideration of the interest that this enriched fraction may have in the food, beverage, and cosmetic industry, its biological effects were further evaluated.

### 3.2. Evaluation of Macrophage Cell Viability after Cfr-LEO Exposure

To obtain a comprehensive understanding of the doses to be further used, we carried out two complementary assays aimed at evaluating both cell viability and cell death. 

**Figure 1:**
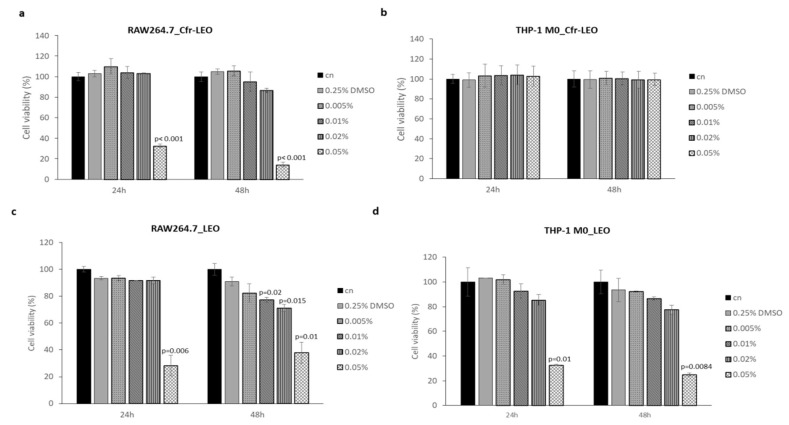
Evaluation of macrophage cell viability after Cfr-LEO and LEO exposure. (**a**) RAW264.7 and (**b**) THP-1 M0 cell viability was measured by MTT assay after 24 and 48 h of treatment with different concentrations of Cfr-LEO (0.005%, 0.01%, 0.02%, 0.05%). The values were plotted as the percentage of cell viability versus untreated cells (cn). Values are the mean ± SD of two biological replicates, each carried out in technical quadruplicates. The statistical significance of the differences between two groups (cells treated with 0.05% of Cfr-LEO *Vs* cn) was analyzed using a two-tailed Student’s *t*-test. (**c**) RAW264.7 and (**d**) THP-1 M0 cell viability were measured by MTT assay after 24 and 48 h of treatment with different concentrations of LEO (0.005%, 0.01%, 0.02%, 0.05%). The values were plotted as the percentage of cell viability versus untreated cells (cn). Values are the mean ± SD of two biological replicates, each carried out in technical triplicates. The statistical significance of the differences between the two groups (cells treated with LEO *Vs* cn) was analyzed using a two-tailed Student’s *t*-test.

The results in Figure 1a,b showed that cell treatment with 0.005%, 0.01%, and 0.02% of Cfr-LEO for 24 and 48 h did not affect the RAW264.7 cell viability; instead, a significant decrease in RAW264.7cell viability after treatment with 0.05% for 24 and 48 h was observed at both time points. No significant decrease in THP-1 M0 cells viability after treatment for 24 and 48 h with all concentrations of Cfr-LEO tested was observed. At the same experimental conditions, whole LEO treatment affected both murine and human macrophage cell viability in a dose and time-dependent manner (Figure 1c,d).

Overall, the results indicate that Cfr-LEO did not exhibit any effect on cell viability at 0.005%, 0.01%, and 0.02% concentrations for both cell lines tested; interestingly, on human macrophages, the same compound can be used even at higher concentrations, up to 0.05%. Therefore, the concentrations of Cfr-LEO ranging from 0.005% to 0.02% were employed for subsequent experiments. 

### 3.3. Evaluation of Macrophage Cytotoxicity after EO Exposure

**Figure 2:**
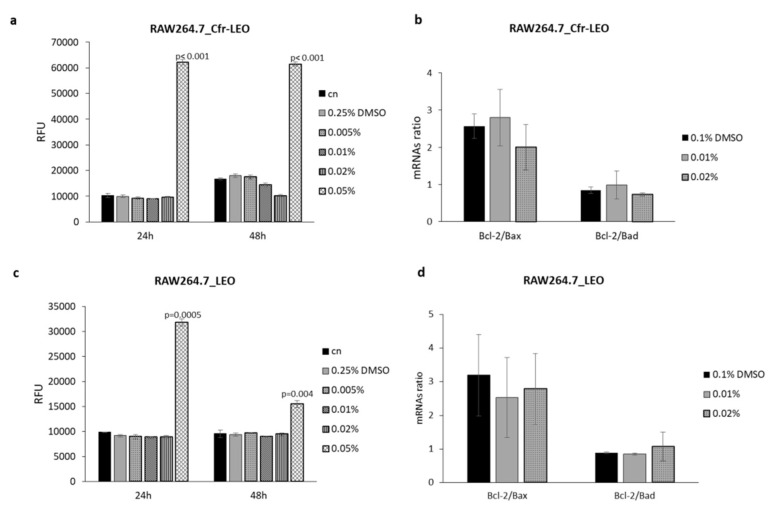
Evaluation of murine macrophage cytotoxicity after Cfr-LEO and LEO exposure. (**a**) RAW264.7 cell cytotoxicity was measured by CellTox Green Cytotoxicity Assay after 24 and 48 h of treatment with different concentrations of Cfr-LEO (0.005%, 0.01%, 0.02%, 0.05%). Values are plotted as Relative Fluorescence Unit (RFU). Values are the mean ± SD of two biological replicates, each carried out in technical duplicates. The statistical significance of the differences between two groups (cells treated with 0.05% of Cfr-LEO *Vs* cn) was analyzed using a two-tailed Student’s *t*-test. (**b**) The effects of Cfr-LEO treatment (0.01%, 0.02%) on Bcl-2, Bax and Bad transcription were assessed by qRT-PCR. Data are represented as Bcl-2/Bax and Bcl-2/Bad ratio. Values are the mean ± SD of two biological replicates. (**c**) RAW264.7 cell cytotoxicity was measured by CellTox Green Cytotoxicity Assay after 24 and 48 h of treatment with different concentrations of LEO (0.005%, 0.01%, 0.02%, 0.05%). Values are plotted as Relative Fluorescence Unit (RFU). Values are the mean ± SD of two biological replicates, each carried out in technical triplicates. The statistical significance of the differences between two groups (cells treated with 0.05% LEO *Vs* cn) was analyzed using a two-tailed Student’s *t*-test. (**d**) The effects of LEO treatment (0.01%, 0.02%) on Bcl-2, Bax, and Bad transcription were assessed by qRT-PCR. Data are represented as Bcl-2/Bax (two biological replicates) and Bcl-2/Bad (three biological replicates) ratio.

THP-1 M0 and RAW264.7 cell lines changes in membrane integrity, that occur as a result of cell death, were measured to exclude cytotoxic effects of Cfr-LEO. Cytotoxicity of RAW264.7 cell lines after incubation for 24 and 48 h with different concentrations of Cfr-LEO (0.005%, 0.01%, 0.02%, 0.05%) was evaluated, and the obtained data are presented in Figure 2a. Data showed that treatment with 0.005%, 0.01%, and 0.02% for 24 and 48 h did not exhibit cytotoxic effects on RAW264.7 cell lines, while, consistent with cell viability assays, a significant increase in RAW264.7 cells toxicity was observed after exposure with 0.05%. 

Several molecular factors such as Bcl-2, Bax, and Bad play a key role in the execution of cells apoptosis [42]. Therefore, Bcl-2, Bax, and Bad gene expression were determined at the transcriptional level in RAW264.7 cell line after incubation for 48 h with different concentrations (0.01% and 0.02%) of Cfr-LEO. In particular, apoptotic gene expression was measured based on Bcl-2/Bax and Bcl-2/Bad ratio, and the obtained data are presented in Figure 2b. No modulation in the Bcl-2/Bax and Bcl-2/Bad ratio was observed in RAW264.7 cells treated with both concentrations of Cfr-LEO tested. We observed a comparable effect of whole LEO on RAW264.7 cell toxicity induction at 0.05% treatment (Figure 2c), as well as on apoptotic gene expression level (Figure 2d). 

**Figure 3:**
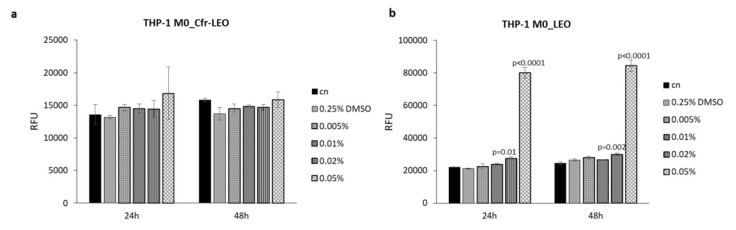
Evaluation of human macrophage cytotoxicity after Cfr-LEO and LEO exposure. (**a**) THP-1 M0 cell cytotoxicity was measured by CellTox Green Cytotoxicity Assay after 24 and 48 h of treatment with different concentrations of Cfr-LEO (0.005%, 0.01%, 0.02%, 0.05%). Values are plotted as the Relative Fluorescence Unit (RFU). Cn: untreated cells. Values are the mean ± SD of two biological replicates, each carried out in technical duplicates. (**b**) THP-1 M0 cell cytotoxicity was measured by CellTox Green Cytotoxicity Assay after 24 and 48 h of treatment with different concentrations of LEO (0.005%, 0.01%, 0.02%, 0.05%). Values are plotted as the Relative Fluorescence Unit (RFU). Cn: untreated cells. Values are the mean ± SD of two biological replicates, each carried out in technical triplicates.

The cytotoxic effects of Cfr-LEO and whole LEO were further evaluated on THP-1 M0 cells. Interestingly, no significant increase in THP-1 M0 cells cytotoxicity after treatment for 24 and 48 h with all concentrations of Cfr-LEO tested was observed (Figure 3a), while whole LEO treatment significantly induced THP-1 cell toxicity at 0.02% and 0.05% at both time point analyzed (Figure 3b).

Overall, these results, in line with the data on cell viability, confirmed that Cfr-LEO did not exhibit any cytotoxic effect at 0.005%, 0.01%, and 0.02% for both cell lines tested. Therefore, these doses were selected for further analysis.

### 3.4. Protective Effects of Cfr-LEO on LPS-Activated Macrophages

The de-regulation of inflammatory homeostasis, in favor of its over-regulation, along with the increase in the pro-inflammatory cytokines is associated with the occurrence of age-related diseases and aging itself [43,44,45]. This phenomenon is known as “Inflammaging” [46,47] and is correlated to the increase of TNF-α, IL-1β, and IL-6. Therefore, enhancing the anti-inflammatory response of the organism is critical for healthy aging. 

*Citrus* EOs have largely been described for their anti-inflammatory properties [22,23,48] often related to the ability to induce a reduction of TNFα, IL1β, and IL6 levels [22,49]. *Citrus medica* EO, mostly containing limonene and γ-terpinene, reduced LPS-induced pro-inflammatory cytokines in murine macrophages; these effects occur simultaneously with the inhibition of the transcription factor NF-kB [49].

Macrophages play a key role in both specific and non-specific immune responses during inflammation [50]. Following activation by LPS, several pro-inflammatory cytokines are secreted and oxidative stress induced. Overproduction of these intermediaries is involved in different inflammatory diseases and cancer [51,52], indicating that the inhibition of macrophage activation could be an important target for inflammatory disease treatment. 

**Figure 4:**
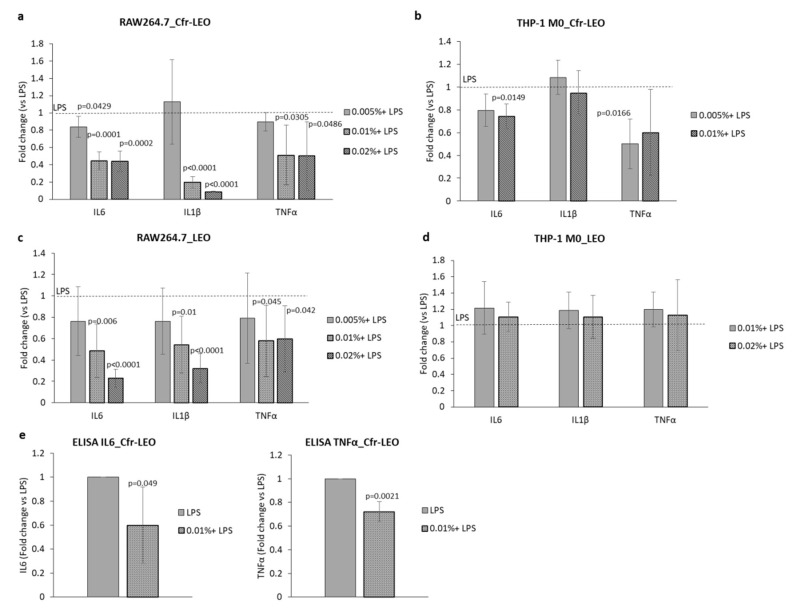
Anti-inflammatory effects of Cfr-LEO and LEO on LPS-activated macrophages. The anti-inflammatory effects of Cfr-LEO and LEO treatment on IL6, IL1b, and TNFα transcription levels were assessed by qRT-PCR analyses. (**a**) RAW264.7 cells were treated for 2 h with Cfr-LEO (0.005%, 0.01%, 0.02%) and then exposed to LPS for 6 h. Values are the mean ± SD of three biological replicates. (**b**) THP-1 M0 were treated for 2 h with Cfr-LEO (0.005%, 0.01%) and then exposed to LPS for 6 h. Values are reported as Fold change versus cells treated with LPS (dashed line). Values are the mean ± SD of three biological replicates for IL6 and TNFα of five biological replicates for IL1β. The statistical significance of the differences between two groups (cells treated with LPS *Vs* cells pre-treated with Cfr-LEO + LPS) was analyzed using a two-tailed Student’s *t*-test. (**c**) RAW264.7 cells were treated for 2 h with LEO (0.005%, 0.01%, 0.02%) and then exposed to LPS for 6 h. (**d**) THP-1 M0 were treated for 2 h with LEO (0.005%, 0.01%) and then exposed to LPS for 6 h. Values are reported as Fold change versus cells treated with LPS (dashed line). Values are the mean ± SD of three to six biological replicates The statistical significance of the differences between two groups (cell treated with LPS *Vs* cells pre-treated with LEO + LPS) was analyzed using a two-tailed Student’s *t*-test. (**e**) IL6 and TNFα protein levels were measured by ELISA in the conditioned medium of RAW264.7 cells treated for 2 h with Cfr-LEO (0.01%) and then exposed to LPS for 6 h. Values are plotted as Fold change versus cell treated with LPS. Values are the mean ± SD of three biological replicates. The statistical significance of the differences between the two groups (cells treated with LPS *Vs* cells pre-treated with Cfr-LEO + LPS) was analyzed using a one-tailed Student’s *t*-test.

To investigate whether Cfr-LEO exhibits protective effects against LPS-induced macrophage activation, we analyzed the effects of Cfr-LEO on the modulation of inflammatory mediators (IL6, IL1β, and TNFα) at the transcriptional level as well as on the GSH/GSSG ratio. In particular, as shown in Figure 4a, the pre-treatment of RAW264.7 for 2 h with each concentration of Cfr-LEO (0.005%, 0.01%, 0.02%), before their exposure to LPS for 6 h, significantly inhibited the production of pro-inflammatory mediators (IL6, IL1β, and TNFα) compared to the 6-h LPS-treated cells. Similarly, the pre-treatment of THP-1 M0 with Cfr-LEO (0.005%, 0.01%) inhibited the production of LPS-induced pro-inflammatory mediators IL6 and, TNFα, while we did not observe a reduction of IL1β Figure 4b. We observed a comparable effect of whole LEO in inhibiting the expression of inflammatory mediators in LPS-induced murine macrophages (Figure 4c), while, at the same doses whole LEO is not effective on human THP-1 macrophages (Figure 4d). The reduction of IL6 and TNFα in RAW264.7 pre-treated with 0.01% of Cfr-LEO, before exposure to LPS, was also confirmed at the protein level (Figure 4e). 

Overall, these data demonstrated that the pre-treatment of macrophages with Cfr-LEO reduces the expression of the pro-inflammatory cytokines TNF-α, IL-1β, and IL-6 induced by LPS. Interestingly, since the assays we have described were performed by using both Cfr-LEO and whole LEO, we can conclude that the enrichment in citral does not alter the well-known anti-inflammatory properties of the whole LEO [22,49].

According to the literature, chronic inflammation is associated with the phenomenon of oxidative stress, caused by the imbalance between the production of reactive oxygen species and the activities of defense systems [53]. EOs are described as natural antioxidant compounds since they exert protective effects by increasing the cellular GSH levels [54] and the GSH/GSSG ratio [55]. In view of these effects, the combined administration of EOs with drugs already used in clinics may reduce the toxic effects produced by therapeutics. For example, EOs from fennel, clove, and cumin reduced the hepatotoxic effects of cyclophosphamide also by increasing GSH levels [54].

**Figure 5:**
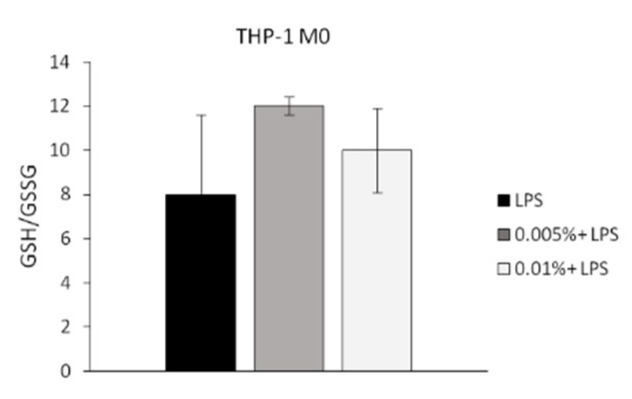
Anti-oxidant effect of Cfr-LEO. The anti-oxidant effects of Cfr-LEO treatment (0.005%, 0.01%) was evaluated by GSH/GSSG-Glo Assay. THP-1 M0 were treated for 2 h with Cfr-LEO (0.005%, 0.01%) and then exposed to LPS for 6 h. Values are expressed as the GSH/GSSG ratio, calculated as follows: GSH/GSSG ratio = (Net total glutathione RLU−Net GSSG RLU)/(Net GSSG RLU/2). Values are the mean ± SD of 2 independent experiments.

Here we also evaluated the capability of Cfr-LEO to contrast the oxidant effects of LPS in human macrophages by performing the GSH/GSSG assay. GSH/GSSG is considered a key indicator of oxidative stress [56,57]. In particular, the increase in the ratio indicates a decrease in cellular oxidative stress [58,59,60]. As shown in Figure 5, we found that in LPS-treated THP1 M0 cells the pre-treatment for 2 h with Cfr-LEO induced the increase of the GSH/GSSG ratio compared to no pre-treated cells, indicating the ability of Cfr-LEO to counteract the LPS-induced oxidative stress. 

Overall, these results suggest that Cfr-LEO can elicit anti-inflammatory and anti-oxidant activities in LPS-stimulated macrophages, providing a rationale to consider its potential role of protective agent against inflammation stimuli.

## 4. Conclusions

To date, the interest of the nutraceutical industry is to use natural compounds that possess both biological and organoleptic properties, conferred for example by citral enrichment. Therefore, in this study, we have selected and mixed four fractions with analogous composition, showing enrichment in citral, and we highlighted their beneficial properties.

In conclusion, we demonstrated that the mix of the four citral-enriched fractions of lemon essential oil (Cfr-LEO), reduces the expression of pro-inflammatory mediators and decreases oxidative stress in murine as well as human macrophage cells. Therefore, having evaluated its non-toxicity and beneficial properties, we suggest that Cfr-LEO, which can certainly be applied in the agri-food industry because of its organoleptic properties, can also represent a preventive tool for improving human health.

## Figures and Tables

**Table 1:** Oligonucleotides used in Real-Time PCR analysis

Gene	Forward	Reverse
Murine		
GAPDH	CCCAGAAGACTGTGGATGG	CAGATTGGGGGTAGGAACAC
BCL-2	GGACTTGAAGTGCCATTGGT	AGCCCCTCTGTGACAGCTTA
BAX	CTGCAGAGGATGATTGCTGA	GATCAGCTCGGGCACTTTAG
BAD	GAGTCGCCACAGTTCGTACC	GGTCCCATCGCACCTAACG
IL6	GAGGATACCACTCCCAACAGACC	AAGTGCATCATCGTTGTTCATACA
TNFα	CACGTCGTAGCAAACCACCAAGTGGA	TGGGAGTAGACAAGGTACAACCC
IL1β	CAACCAACAAGTGATATTCTCCATG	GATCCAACACTCTCCAGCTGCA
Human		
GAPDH	ATGGGGAAGGTGAAGGTCG	GGGTCATTGATGGCAACAATAT
IL6	GGTACATCCTCGACGGCATCT	GTGCCTCTTTGCTGCTTTCAC
TNFα	CCAGGCAGTCAGATCATCTTCTC	AGCTGGTTATCTCTCAGCTCCAC
IL1β	ACAGATGAAGTGCTCCTTCCA	GTCGGAGATTCGTAGCTGGAT

**Table 2:** Volatile compounds identified in winter lemon essential oil (EO) (whole LEO) and in the selected fractions (Fr13, Fr14, Fr15, Fr16).

No	VOLATILE COMPOUND	WHOLE LEO		Fr13		Fr14		Fr15		Fr16	
		Rt ^a^	Area (%) ^b^	Rt	Area (%)	Rt	Area (%)	Rt	Area (%)	Rt	Area (%)
1	α-Thujene	1.688	0.43	1.674	0.42	1.676	0.42	1.675	0.42	1.675	0.42
2	α-Pinene	1.757	1.81	1.743	1.79	1.745	1.79	1.744	1.79	1.744	1.79
3	Camphene	1.892	0.06	1.875	0.05	1.878	0.06	1.876	0.05	1.877	0.05
4	Sabinene	2.132	1.83	2.112	1.81	2.115	1.81	2.113	1.81	2.114	1.81
5	β-Pinene	2.197	10.88	2.178	10.81	2.181	10.80	2.18	10.80	2.18	10.79
6	Myrcene	2.309	1.57	2.286	1.56	2.29	1.56	2.288	1.56	2.289	1.56
7	Octanal	2.447	0.07	2.419	0.06	2.422	0.06	2.419	0.06	2.421	0.06
8	Phellandrene	2.504	0.05	2.477	0.04	2.481	0.04	2.478	0.04	2.48	0.04
9	δ-3-Carene	2.593	0.01	2.566	0.01	2.572	0.01	2.57	0.01	2.571	0.01
10	α-Terpinene	2.683	0.21	2.654	0.20	2.661	0.20	2.657	0.20	2.657	0.20
11	Limonene	2.982	67.10	2.965	67.09	2.972	67.09	2.971	67.07	2.972	67.05
12	(Z)- β-Ocimene	3.063	0.05	3.008	0.05	3.014	0.05	3.012	0.05	3.013	0.05
13	(E)- β-Ocimene	3.198	0.12	3.163	0.12	3.17	0.12	3.166	0.12	3.168	0.12
14	γ-Terpinene	3.434	9.32	3.404	9.27	3.411	9.27	3.408	9.26	3.41	9.26
15	Cis-Sabinene hydrate	3.556	0.04	3.522	0.01	3.52	0.01	3.513	0.02	3.515	0.03
16	Octanol	3.654	0.01	3.612	0.00	3.609	0.00	3.606	0.00	3.608	0.00
17	Terpinolene	3.936	0.41	3.893	0.41	3.9	0.40	3.895	0.40	3.898	0.40
18	Trans-Sabinene hydrate	4.132	0.03	4.1	-	4.1	-	4.1	-	4.1	-
19	Linalool	4.158	0.13	4.109	0.13	4.115	0.14	4.109	0.15	4.112	0.15
20	Nonanal	4.228	0.11	4.179	0.11	4.185	0.10	4.18	0.10	4.183	0.10
21	Citronellal	5.186	0.10	5.134	0.10	5.14	0.10	5.134	0.10	5.137	0.10
22	Terpinen-4-ol	5.65	0.03	5.596	0.03	5.601	0.03	5.594	0.03	5.598	0.03
23	α-Terpineol	5.917	0.17	5.724	-	5.729	0.01	5.863	0.03	5.865	0.05
24	Decanal	6.223	0.04	6.171	0.03	6.177	0.03	6.17	0.03	6.174	0.03
25	Nerol+Citronellol	6.678	0.05	6.633	0.00	6.629	0.01	6.622	0.02	6.625	0.03
26	Neral	6.905	1.00	6.854	1.35	6.86	1.33	6.854	1.31	6.858	1.31
27	Geraniol	7.192	0.05	7.14	-	7.14	-	7.148	-	7.147	0.01
28	Geranial	7.483	1.72	7.435	2.19	7.442	2.21	7.437	2.21	7.44	2.21
29	Undecanal	8.15	0.02	8.096	0.02	8.102	0.02	8.096	0.02	8.1	0.02
30	Methyl geranate	8.682	0.02	8.628	0.03	8.634	0.03	8.628	0.03	8.632	0.03
31	Citronellyl acetate	9.006	0.03	8.951	0.03	8.956	0.03	8.951	0.03	8.955	0.03
32	Neryl acetate	9.203	0.46	9.15	0.51	9.155	0.49	9.15	0.49	9.154	0.48
33	Geranyl acetate	9.54	0.31	9.486	0.35	9.491	0.33	9.486	0.33	9.49	0.33
34	β-Caryophyllene	10.105	0.23	10.05	0.23	10.056	0.23	10.051	0.23	10.055	0.23
35	Bergamotene	10.409	0.31	10.357	0.32	10.362	0.31	10.357	0.32	10.361	0.32
36	Valencene	11.325	0.02	11.275	0.02	11.279	0.02	11.276	0.02	11.279	0.02
37	Bicyclogermacrene	11.374	0.05	11.325	0.04	11.328	0.05	11.325	0.04	11.328	0.04
38	(Z)-α-Bisabolene	11.475	0.05	11.432	0.04	11.435	0.04	11.432	0.04	11.435	0.04
39	β-Bisabolene	11.553	0.48	11.512	0.48	11.516	0.48	11.513	0.48	11.515	0.48
40	γ-Elemene	11.931	0.02	11.896	0.02	11.899	0.02	11.896	0.02	11.898	0.02
41	2-Norbornarol	12.859	0.02	12.83	0.02	12.832	0.02	12.831	0.02	12.831	0.02
42	Campherenol	12.947	0.02	12.919	0.02	12.92	0.02	12.919	0.02	12.92	0.03
43	Bisabolol	13.046	0.02	13.02	0.03	13.02	0.03	13.02	0.03	13.02	0.03

^a^ Rt: Retention time in minutes ^b^ Area (%): percentage obtained by FID peak-area normalization. The values reported in the table are representative of one of the three replicates.

**Table 3:** The percentage trend of the main chemical classes of identified in winter lemon EO (whole LEO) and its fractions (Fr13, Fr14, Fr15, Fr16). Citral (in red) is more concentrated in the fractions than in the starting whole EO. The values reported in the table are representative of one of the three replicates.

Chemical Classes	Whole LEO (%)	Fr 13 (%)	Fr 14 (%)	Fr 15 (%)	Fr 16 (%)
Monoterpenes	93.9	93.65	93.64	93.61	93.6
Total Aldehydes	3.06	3.87	3.85	3.84	3.84
citral (geranial + neral)	2.73 (1.72 + 1)	3.54 (2.19 + 1.35)	3.53 (2.21 + 1.33)	3.52 (2.21 + 1.31)	3.52 (2.21 + 1.31)
Aldehydes minus citral	0.33	0.33	0.32	0.32	0.32
Sesquiterpenes	1.16	1.15	1.15	1.15	1.15
Esters	0.82	0.92	0.88	0.87	0.87
Aliphatic Alcohols	0.43	0.17	0.20	0.24	0.28
Sesquiterpene Alcohols	0.06	0.07	0.07	0.07	0.07

## Supplementary Materials

The following are available online at https://www.mdpi.com/2304-8158/9/9/1290/s1, Figure S1: Evaluation of the reproducibility of the fractionation process. (**a**) The analysis of the coefficient of variation (CV %) for the three replicates. (**b**) Graph showing the percentage of each compound in the four examined fractions. Each point represents the mean ±SD of the three independent replicates of the fractionation process. (**c**) The histogram represents the mean ±SD of the percentage of citral in the selected fractions. Values are the mean of three independent replicates of the fractionation process.
