# Supplementary material for: Biological Properties of a Citral-Enriched Fraction of Citrus limon Essential Oil
Source: Foods. 2020 Sep 14;9(9):1290. doi: 10.3390/foods9091290 (PMC7555671; doi:10.3390/foods9091290)
Supplement: Supplementary file 1 [file foods-09-01290-s001.pdf]

foods

MDPI

Supplemenatry Materials

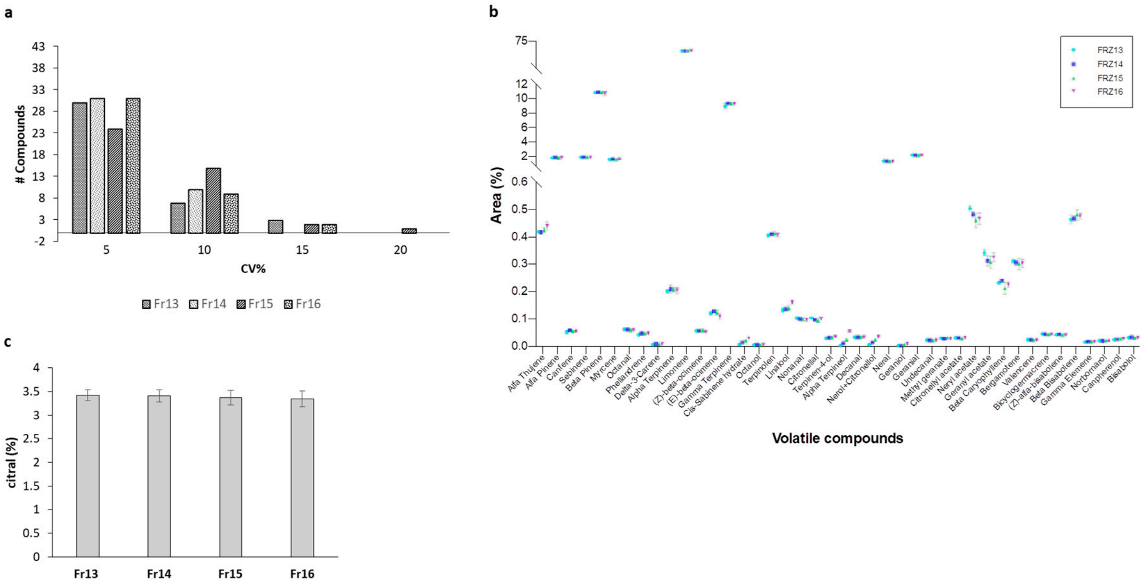

Figure S1. Evaluation of the reproducibility of the fractionation process. (**a**) The analysis of the coefficient of variation (CV %) for the three replicates. (**b**) Graph showing the percentage of each compound in the four examined fractions. Each point represents the mean ±SD of the three independent replicates of the fractionation process. (**c**) The histogram represents the mean ±SD of the percentage of citral in the selected fractions. Values are the mean of three independent replicates of the fractionation process.
